# Comparisons between intragastric and small intestinal delivery of enteral nutrition in the critically ill: a systematic review and meta-analysis

**DOI:** 10.1186/cc12800

**Published:** 2013-06-21

**Authors:** M Deane Adam, Dhaliwal Rupinder, G Day Andrew, J Ridley Emma, R Davies Andrew, K Heyland Daren

**Affiliations:** 1 Intensive Care Unit, Royal Adelaide Hospital, Adelaide, SA, Australia; 2 Discipline of Acute Care Medicine, University of Adelaide, Adelaide, SA, Australia; 3 Clinical Evaluation Research Unit, Kingston General Hospital, Kingston, ON, Canada; 4 Department of Epidemiology and Preventive Medicine, Monash University, Melbourne, VIC, Australia

## Abstract

**Introduction:**

The largest cohort of critically ill patients evaluating intragastric and small intestinal delivery of nutrients was recently reported. This systematic review included recent data to compare the effects of small bowel and intragastric delivery of enteral nutrients in adult critically ill patients.

**Methods:**

This is a systematic review of all randomised controlled studies published between 1990 and March 2013 that reported the effects of the route of enteral feeding in the critically ill on clinically important outcomes.

**Results:**

Data from 15 level-2 studies were included. Small bowel feeding was associated with a reduced risk of pneumonia (Relative Risk, RR, small intestinal vs. intragastric: 0.75 (95% confidence interval 0.60 to 0.93); *P *= 0.01; I^2 ^= 11%). The point estimate was similar when only studies using microbiological data were included. Duration of ventilation (weighted mean difference: -0.36 days (-2.02 to 1.30); *P *= 0.65; I^2 ^= 42%), length of ICU stay (WMD: 0.49 days, (-1.36 to 2.33); *P *= 0.60; I^2 ^= 81%) and mortality (RR 1.01 (0.83 to 1.24); *P *= 0.92; I^2 ^= 0%) were unaffected by the route of feeding. While data were limited, and there was substantial statistical heterogeneity, there was significantly improved nutrient intake via the small intestinal route (% goal rate received: 11% (5 to 16%); *P *= 0.0004; I^2 ^= 88%).

**Conclusions:**

Use of small intestinal feeding may improve nutritional intake and reduce the incidence of ICU-acquired pneumonia. In unselected critically ill patients other clinically important outcomes were unaffected by the site of the feeding tube.

## Introduction

In the critically ill, nutritional therapy is a component of standard care. Delivery into the gastrointestinal tract is the preferred route of nutrient administration, via a tube into either the stomach or small intestine [1]. However, whether these feeding tubes should be preferentially placed into the stomach or small intestine remains contentious. Several clinical practice guidelines have recommended that enteral nutrition should be commenced using an intragastric tube [1,2], whereas other guidelines advise that when small bowel feeding is feasible that this route is preferable [3].

The advantages of commencing with intragastric feeding include that naso- or oro-gastric tubes are relatively easy to insert, so that once a decision is made to feed, delivery of nutrient can promptly commence. However, disadvantages of the intragastric approach include delayed gastric emptying, which occurs frequently in the critically ill [4,5], and predisposes to inadequate nutrient administration. Indeed, observational studies, in which most patients receive intragastric feeding, have shown that the proportion of calories and protein delivered to critically ill patients are about 50 to 70% of targeted calorie and protein loads [6-8].

Small intestinal feeding tubes are more difficult to insert, often requiring specific expertise and equipment. Their potential advantages include bypassing the stomach, which should theoretically 'guarantee' delivery of nutrients, as the major gastrointestinal motility disorders in the critically ill appear to occur in the antral-pyloro region of the stomach [9]. Not only does increasing administration of nutrients have the potential to reduce mortality and length of stay, particularly in those patients at risk of critical illness malnutrition and its consequences [10], but delivery of nutrients into the small intestine has been reported to reduce the incidence of hospital-acquired pneumonia, possibly because episodes of gastro-oesophageal regurgitation occur less frequently [11,12]. Intuitively, a reduction in pneumonia should shorten intensive care unit (ICU) and hospital length of stay and may reduce mortality [13].

Systematic reviews and meta-analysis have been published that evaluated small bowel and gastric feeding in the critically ill [14-17]. An update incorporating important recent studies [18-20] was published by Jiyong and colleagues [17]. However, a limitation of the latter review is that the authors included a study in which patients received care on the general hospital ward rather than a critical care environment. In addition, Jiyong and colleagues included studies of both adult and paediatric subjects. Finally, data from the ENTERIC study were not included, and we believe that this study is the pivotal study in the area [21]. For these reasons we sought to update previous reviews to determine whether small bowel, when compared to intragastric delivery of nutrition, is associated with improved outcomes in the critically ill.

The objectives of this study were to determine the effects of small bowel and gastric tube delivery of liquid nutrients on hospital-acquired pneumonia, duration of mechanical ventilation, length of ICU and hospital stay, mortality and nutritional intake in adult critically ill patients.

## Methods

The meta-analysis was performed in line with the recommendations from the Preferred Reporting Items for Systematic Reviews and Meta-Analyses (PRISMA) statement [22].

### Study identification

We conducted a systematic review of the published literature to identify all relevant randomised clinical trials. Using text word or MeSH headings containing: "randomized"; "blind"; "clinical trial"; "nutrition"; "enteral"; "small bowel"; "gastric"; "nasojejunal"; "nasoduodenal"; and "nasogastric" computerised searches for relevant articles on MEDLINE, EMBASE, BIIOSIS, CINAHL electronic databases Cochrane Controlled Trials Register from 1990 to March 2013 were performed. Reference lists of review articles and original studies were hand searched and relevant articles extracted.

### Study eligibility criteria

All primary studies were retrieved and reviewed. Primary studies were eligible for inclusion if they: (1) studied adult patients with critical illness; (2) compared small bowel (delivered into the jejunum or duodenum) to gastric delivery; (3) included clinically important outcomes, such as mortality, infectious complications (including hospital-acquired pneumonia), length of stay or major nutritional endpoints; and (4) were randomised clinical trials (RCTs).

We defined patients with critical illness as those who were cared for in a critical care environment. Utilizing a scoring system that has been previously reported [23], RCTs were rated for methodological quality. Using previously piloted forms [23], two reviewers independently, and in duplicate, then abstracted data from these studies. Agreement was reached by consensus. We attempted to contact the authors of included studies and requested further information not contained in published articles.

### Data synthesis

The primary outcome was the incidence of ICU-acquired pneumonia. Secondary outcomes were duration of mechanical ventilation, duration of ICU stay, duration of hospital stay, mortality, and nutritional intake. We used definitions of ICU-acquired pneumonia as defined by the original study investigators.

To quantify nutritional intake we included only studies that reported the mean (SD) percentage of calories or volume delivered when compared to the patients' energy expenditure or prescribed volume as estimated by weight-based or complex calculation. Data from all studies were combined to estimate the common risk ratio (RR) and associated 95% confidence intervals (CI) for mortality and hospital-acquired pneumonia. In the meta-analysis, we used maximum likelihood methods of combining risk ratios across all trials and examined the data for evidence of heterogeneity within groups. The Mantel-Haenszel method was used to test the significance of treatment effect. We used a random effects model to estimate the overall relative risk. Heterogeneity was determined using the Chi squared test and interclass correlation I^2^. We also analysed the effect of small bowel delivery on duration of mechanical ventilation, ICU stay and hospital stay; the weighted mean difference (WMD) was used to describe the standardised difference between mean duration of stay from small bowel and intragastric delivery, respectively. *P*-values < 0.05 were considered significant.

### Subgroup and sensitivity analyses

There were substantial limitations when interpreting data from two of the included studies. Taylor and colleagues reported the effect of 'enhanced' enteral nutrition, that ideally was administered via a small intestinal feeding tube [24]. However, only 34% of the patients actually achieved feeding via the small bowel, which is markedly inferior to rates of successful small intestinal tube placement reported by other groups [25]. Minard and colleagues compared patients who received early immune-enhanced enteral nutrition via the small bowel to those receiving delayed immune-enhanced enteral nutrition via the stomach [26]. Accordingly, when indicated, meta-analyses of outcome data were performed with, and without, these two studies.

It should also be recognised that the diagnosis of ICU-acquired pneumonia can be subjective. For this reason, when evaluating the effect of small intestinal feeding on the incidence of pneumonia we also report the subgroup of studies that used microbiological data in association with clinical data [27].

## Results

The literature search resulted in 22 RCTs. After reviewing these studies, four were excluded because they were systematic reviews [14-17], one had < 50% of enrolled patients admitted to an ICU [28], one did not report clinical outcomes [11] and one [29] was an analysis of data already included.

### Studies

Results from these 15 level-2 RCTs were aggregated. None of the studies were able to blind treating health care providers to the route of delivery once patients were randomised, and only three studies included patients from more than one ICU (Kortbeek, *n *= 2; Montejo, *n *= 11; Davies, 2012, *n *= 17). Only two groups reported registration of their study [18,21]. The characteristics of the studies are summarised in Table 1.

**Table 1:** Characteristics of included studies

Study	Total subjects	Population *(exclusion criteria are listed when relevant)*	(i) Intervention *(small intestinal or gastric group allowed gastrokinetic drug/s and type)* (ii) Technique (iii) Success *of small bowel placement in intervention group* (iv) Time *to placement*	(i) Study design (ii) Study registered?	Outcomes (i) primary (ii) secondary	How Pneumonia diagnosed (Blinded + Criteria)	Assessment of Methods (Score^1^)
1. Montecalvo 1992	38	Med/Surg Intensive Care Unit (ICU). Patients anticipated to require ≥ days of nutrition	Small bowel versus Gastric (no comment gastrokinetic drugs) (ii) endoscopy (iii) 14/19 (74%) (iv) 0.3 +/- 0.9 days; mean (SD)	Randomised Control Trial (RCT) Multicenter (2 sites) (ii) No	(i) Administration of nutrient (ii) Pneumonia Mortality Duration of mechanical ventilation (MV) Duration of ICU stay	Blinded New Chest x-ray (CXR) changes + 3 of: (i) sputum > 25 leukocytes < 10 epithelial and numerous bacteria; (ii) sputum > 25 leukocytes < 10 epithelial and nosocomial pathogen present; (iii) Temperature > 101.4° F (38.6°C); OR (iv) White Cell Count > 10,000 (units)	Conceal: Uncertain Intension To Treat (ITT): No (analyzed according to location of feeding tube, rather than intention to treat) Blinding: No (Score 8)

2. Kortbeek 1999	80	Trauma Likely to require mechanical ventilation (MV) > 48 hrs, and enroled < 72 from admission, and Injury Severity Score (ISS) > 16 Exclusion traumatic pancreatitis and physiologic instability precluding transportation for fluoroscopic placement of a duodenal tube	Small bowel versus Gastric (no comment gastrokinetic drugs) (ii) fluoroscopy (iii) not reported (iv) 30 minutes (15 to 120 minutes); med (range)	RCT Multicenter (2 sites) (ii) No	(i) Administration of nutrient (ii) Mortality Pneumonia Duration of MV Duration of ICU stay Duration of hospital stay	Blinded New CXR changes and 2 of: Temp > 38.5°C,; (ii) WCC > 10,000 or < 3,000 (units); (iii) purulent sputum; or (iv) pathogenic bacteria cultured from bronchoalveolar lavage (BAL)	Conceal: Yes ITT: Yes Blinding: No (Score 11)

3. Taylor 1999	82	Traumatic Brain Injury (TBI), MV, Glasgow Coma Scale (GCS) score > 3, and at least one reactive pupil at some time during the first 24 hrs, as well as suitable for EN. Exclusion criteria included presence of any other organ failure	Small bowel versus Gastric (no comment gastrokinetic drugs) (ii) 'blind' (iii) 34% (iv) not reported	RCT Single-center (ii) No	(i) Neurological outcome at 6 months (ii) Mortality (6 months) and pneumonia	Diagnosis of pneumonia not described	Conceal: Uncertain ITT: Yes Blinding: No (Score 10)

4. Kearns 2000	44	Medical ICU, MV and EN ≥ days Excluded patients with pancreatitis and ileus	Small bowel versus Gastric (gastrokinetic drugs allowed but not reported) (ii) blind placement with metoclopramide (iii) 21/21 (100%), but three required fluoroscopy (iv) < 10 minutes except if required fluoroscopy - time not reported in those patients	RCT Single-center (ii) No	(i) Pneumonia (ii) Mortality, duration of ICU and hospital stay, and nutrient administered	Not blinded New CXR changes and and 2 of: Temp > 38.5°C; (ii) WCC > 10,000 or (iii) positive glucose test or blue discolouration in endotracheal secretions.	Conceal: Uncertain ITT: Yes Blinding: No (Score 9)

5. Minard 2000	27	Trauma GCS^7 ^3 to 10 Excluded patients with sepsis, kidney or respiratory failure or requiring vasoconstricting drugs	Small bowel versus Gastric (no comment gastrokinetic drugs) (ii) endoscopy (iii) 13/15 (87%) (iv) not reported	RCT Single-center (ii) No	(i) Length of ICU stay (ii) Mortality, pneumonia duration of MV and hospital stay	Not blinded CXR changes+ Purulent sputum+ Temp > 101°F+ WBC > 12,000 OR BAL > 100,000 CFUs	Conceal: Uncertain ITT: No Blinding: No (Score 7)

6. Esparaza 2001	54	Medical ICU Inclusion criteria not reported	Small bowel versus Gastric (gastrokinetic drugs, erythromycin or metoclopramide- allowed in both groups. Reported as administration per patient-fed days) (ii) electromyographic guided with erythromycin, metoclopramide and/or fluoroscopy (iii) 21/27 (78%) (iv) not reported	RCT Single-center (ii) No	(i) Aspiration events based on radiolabelled 'meal' and gamma camera) (ii) Mortality, administration of nutrient	Pneumonia not reported	Conceal: Uncertain ITT: Yes Blinding: No (Score 8)

7. Boivin 2001	80	Med/Surg ICU, MV in 98% Enteral Nutrition (EN) ≥72 hrs Excluded: pancreatitis, burns, severe head injury	Small bowel versus Gastric (all patients in both groups received erythromycin) (ii) blind placement with erythromycin, with fluoroscopy for failed attempts (iii) 28/40 (71%) (iv) 304 minutes	RCT Single-center (ii) No	(i) Administration of nutrient (ii) Mortality	Pneumonia not reported	Conceal: Uncertain ITT: No Blinding: No (Score 6)

8. Day 2001	25	Primary neurological diagnosis and expected to receive EN for ≥ 3 days Patients were excluded who had gastroparesis	Small bowel versus Gastric (No comment regarding gastrokinetic drugs) (ii) blind placement (iii) not reported (iv) not reported	RCT Single-center (ii) No	(i) Administration of nutrient (ii) Pneumonia	Did not report how pneumonia was diagnosed	Conceal: Uncertain ITT: Yes Blinding: No (Score 5)

9. Davies 2002	73	Med/Surg ICU Expected to receive EN ≥ days	Small bowel versus Gastric (gastrokinetic drugs excluded) (ii) endoscopy (iii) 33/34 (97%) (iv) time to placement not reported. However, time to commencing nutrition was delayed in patients receiving small intestinal feeds (81 vs. 55)	RCT Single-center (ii) No	(i) Intolerance to enteral nutrition (ii) Mortality Pneumonia Duration of ICU stay	Not blinded Clinical criteria, CXR changes and microbiological data	Conceal: Uncertain ITT: No Blinding: No (Score 8)

10. Neumann 2002	60	Medical ICU In need of enteral nutrition excluded gastroparesis, ileus and pancreatitis	Small bowel versus Gastric (no comment gastrokinetic drugs) (ii) blind placement, fluoroscopy as second line - required in 7/20 attempts. (iii) not reported (iv) not reported	RCT Single-center (ii) No	(i) Efficacy of nutrition (ii) Aspiration events	Pneumonia not reported	Conceal: Uncertain ITT: Yes Blinding: No (Score 6)

11. Montejo 2002	101	Mixed ICUs EN > 5 days Patients with gastroparesis allowed to enter	Small bowel versus Gastric (no comment gastrokinetic drugs) (ii) technique depending on local expertise but included endoscopy (*n *= 18), fluoroscopic guidance (*n *= 12), blind technique (*n *= 15), or by echography (*n *= 5). (iii) 100% (iv) time to small intestine 21.0 ± 9.8 vs. gastric 5.3 ± 7.9 hrs	RCT Multi-center (14 ICUs) (ii) No	(i) Pneumonia (ii) Mortality Duration of ICU stay	Not blinded Diagnosed according to criteria described by the Centre for Disease Control	Conceal: Not sure ITT: Yes Blinding: No (Score 6)

12. Hsu 2009	121	Medical ICU EN > 3 days Excluded intractable vomiting, severe diarrhea, paralytic ileus and acute pancreatitis	Small bowel Versus Gastric (gastrokinetic drugs, such as metoclopramide, erythromycin, cisapride, allowed but not routinely administered, administered *n *= 20/62 (32%) gastric and 18/59 (31%) small intestine (ii) blind placement with endoscopy for failed cases (iii) not reported (iv) not reported	RCT Single-center (ii) No	(i) Nutrient administered (ii) Mortality Pneumonia Duration of MV ICU and hospital stay	Blinded New CXR changes and one of: T > 38 or < 36 with no other recognized cause; (ii) WCC > 12,000 or < 4,000; or (iii) for adults ≥ 70 years and altered mental status at least two of new or change n purulent sputum, new cough or tachypnea, worsening gas exchange, and bronchial breath sounds	Conceal: Yes ITT: Yes Blinding: No (Score 9)

13. White 2009	108	Medical ICU MV > 24 hrs	Small bowel versus Gastric (gastrokinetic drugs, metoclopramide and erythromycin, administered for GRVs > 200 mL) (ii) 'blind' with erythromycin (iii) 40/50 (80%) (iv) Not reported	RCT Single-center (ii) Yes (prospective)	(i) Time to reach goal feed rate (ii) Mortality Pneumonia Duration of MV and ICU stay (iii) weight-based	Not blinded New fever Leukocytosis New CXR changes, increased pulmonary secretions and clinical pulmonary infection score (CPIS) > 6	Conceal: Yes ITT: Yes Blinding: No (Score 7)

14. Acosta-Escribano 2010	104	TBI on MV Expect EN required for ≥ 5 days Glasgow coma scale (GCS) < 9, APACHE II between15-30, sequential organ failure assessment (SOFA) < 6	Small bowel Versus Gastric (metoclopramide administered for two consecutive GRV > 200 mL) (ii) blind or fluoroscopy (iii) 47/50 (94%) (iv) not reported	RCT Single-center (ii) No	(i) Pneumonia (ii) Administration of nutrient Mortality (30 day) Pneumonia Duration of MV, ICU and hospital stay	Not blinded CPIS > 6 required for diagnosis. However, microbiological data collected in all patients and only one patient diagnosed with pneumonia did not have a pathogen isolated from the lower respiratory tract	Conceal: No ITT: Yes Blinding: No (Score 9)

15. Davies 2012	181	Mixed ICUs within 72 hrs of admission Receiving MV Receiving opiate drug via infusion Gastric residual volume (GRV) > 150 ml or > 500 ml over 12 hrs	Small bowel versus Gastric (metoclopramide ≥ erythromycin prn) (ii) Self-migrating + erythromycin (iii) 79/92 (87%) (iv) 15 (7 to 32) hours; median (IQR)	RCT Multi-center (17 sites) (ii) Yes (prospective)	(i) Energy delivery (ii) Mortality Pneumonia Duration of MV, ICU and hospital stay	Blinded Consensus panel of three clinicians, pneumonia diagnosed by at least two members based on temp, WCC, sputum, P/F ratio, microbiological results and CXR	Conceal: Yes ITT: Yes Blinding: No (Score 11)

(^1 ^Score calculated as described [23]).

### Placement of small intestinal feeding catheters

Various techniques were used to insert small intestinal tubes (Table 1).

Success - and time to successful placement - of small intestinal feeding tubes were not reported in all studies. When reported (*n *= 11), success rates varied between 34 and 100%, with the median time to placement ranging from 5 hours to 1.5 days (Table 1). Repeated testing to confirm that the feeding tube remained in either the stomach or small intestine throughout the patient's admission was not reported in any study.

### Standardisation in the intragastrically-fed group

While the 'control' group in all studies was, at least initially, intragastric delivery, the use of gastrokinetic drugs was inconsistently reported (Table 1).

### Outcomes

#### Pneumonia

Twelve studies reported the incidence of ICU-acquired pneumonia. The reported incidence of pneumonia ranged from 9% to 46% of patients studied. Pneumonia was diagnosed according to a variety of techniques (Table 1), with six studies incorporating microbiological and clinical data [20,21,30-33], four studies used clinical signs and radiological changes [18,19,34,35] and two studies did not describe the technique used to make the diagnosis of pneumonia [24,36]. In only four studies were the investigators blinded to treatment allocation when making the diagnosis of pneumonia [19,21,30,31].

**Figure 1:**
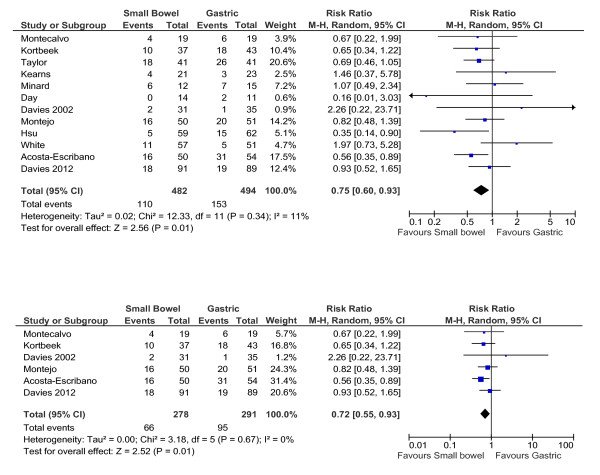
**Small intestinal feeding and pneumonia**. Twelve studies reported the: (**A**) incidence of pneumonia with (**B**) six studies incorporating both microbiological data with clinical data when making the diagnosis.

Small bowel feeding was associated with a reduced risk of ICU-acquired pneumonia when compared to gastric (relative risk (RR): small intestine vs. intragastric: 0.75 (0.60 to 0.93) *P *= 0.01; test for heterogeneity I^2 ^= 11%; Figure 1A). The point estimate was unaffected when the studies by Taylor and Minard were removed (RR: 0.75 (0.56 to 1.00); *P *= 0.05; I^2 ^= 21%). When analysing only studies that included microbiological diagnosis, these results remained similar (RR: 0.72 (0.55 to 0.93); *P *= 0.01; I^2 ^= 0%; Figure 1B).

#### Length of stay

**Figure 2:**
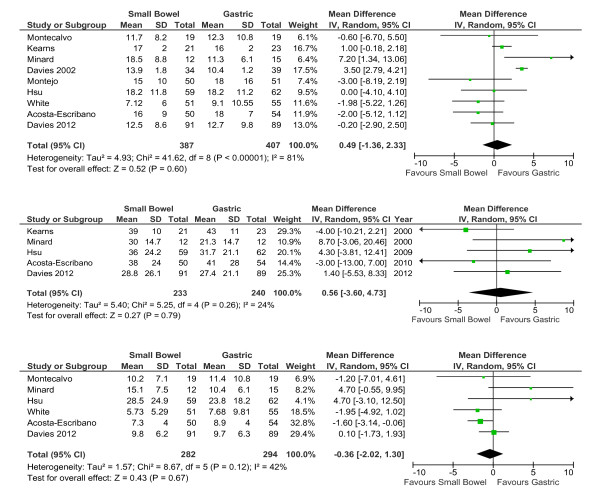
**Small intestinal feeding and duration of ICU- and hospital admission and mechanical ventilation**. (**A**) Nine studies reported the duration of admission into the Intensive Care Unit (ICU), (**B**) five studies reported hospital admission length-of-stay, and (**C**) six reported length of mechanical ventilation.

Nine studies reported on ICU length of stay. Although heterogeneity was present, length of stay appeared unaffected regardless of whether small intestine or intragastric tubes were used (weighted mean difference (WMD): 0.49 days (-1.36 to 2.33); *P *= 0.60; I^2 ^= 81%; Figure 2A). Results were unchanged when the study by Minard was excluded (WMD: 0.04 days (-1.85 to 1.93); *P *= 0.97; I^2 ^= 82%). Hospital length of stay was also unaffected by small bowel or intragastric administration of nutrients in the five studies that reported this outcome (WMD: 0.56 days (-3.60 to 4.73) *P *= 0.79; I^2 ^= 24%; Figure 2B).

#### Duration of mechanical ventilation

Administration of nutrients directly into the small intestine did not appear to influence duration of mechanical ventilation (WMD: -0.36 (-2.02 to 1.30); *P *= 0.67; I^2 ^= 42%; Figure 2C).

#### Mortality

**Figure 3:**
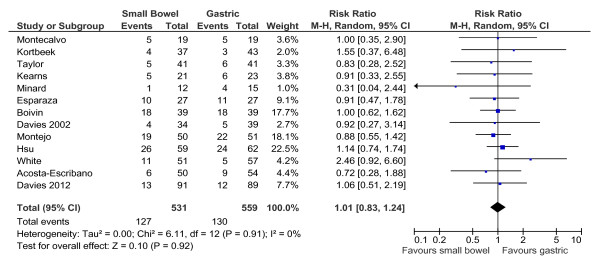
**Small intestinal feeding and mortality**. **Thirteen studies reported mortality data**.

Thirteen studies reported mortality data. Feeding via small intestinal or intragastric tube did not affect mortality (RR: 1.01 (95% CI: 0.83 to 1.24); *P *= 0.92; I^2 ^= 0%; Figure 3). When the studies by Taylor and Minard were excluded, data were unchanged (RR: 1.03 (0.84 to 1.27); *P *= 0.78; I^2 ^= 0%).

#### Nutritional administration

Targets for nutritional delivery were based on formulae (*n *= 7) or weight-based calculations (*n *= 4), with a few studies either not, or inadequately, reporting how the nutrition target was derived (*n *= 4) (Table 2). Only eight studies reported the number of days of enteral nutrition provided, which varied from less than 4 days to more than 11 days (Table 2).

**Table 2:** Nutritional outcomes reported

Study	Days of artificial nutrition	Determination of energy requirements	Reported nutritional intake such that data could be incorporated into meta-analysis?
1. Montecalvo 1992	Gastric 10.3 ± 10.0 d and Small intestine 10.4 ± 7.4 d	Inadequately described	Yes

2. Kortbeek 1999	Not reported	Harris-Benedict	No, did not report calories received but time to tolerate feeds for 24 consecutive hours

3. Taylor 1999	Control 11 days vs. Intervention 9 days (median).	Schofield	No, but intervention patients had a higher mean percentage of energy

4. Kearns 2000	Gastric 8 ± 1 days and Small intestine 9 ± 1 mean ± SEM	Calculated energy expenditure; calculation not specified	Yes

5. Minard 2000	Not reported	Weight-based	No, as day of commencing nutrition different

6. Esparaza 2001	Gastric 4.1 d Small intestine 3.6 d; (mean)	Harris Benedict	No, spread of data not reported

7. Boivin 2001	Not reported	Weight-based	No, data only in graphs and not described in text

8. Day 2001	Not reported	Harris-Benedict	Not included as data presented as percentage target per day. However, increased nutrient delivery was observed with gastric feeding on days 2 and 3 but not afterward.

9. Davies 2002	Gastric 8.2 d Small intestine 8.6 d; mean	Harris Benedict	No, data for calories delivered in first 48 hours, but not thereafter.

10. Neumann 2002	Gastric 6.5 ± 4.4 d Small intestine 5.3 ± 4.5 d Mean ± SD	Not described	No, but time to reach goal reported

11. Montejo 2002	Gastric 12 ± 10 d Small intestine 11 ± 8 d Mean ± SD	Not standardised but determined by each site investigator	Yes

12. Hsu 2009	Not reported	Ireton-Jones equation	Yes

13. White 2009	Gastric 3.92 (1.05 to 7.88) vs. small bowel 3.63 (1.89 to 6.92) days; median (IQR)	Weight based	No, reported as energy deficit with energy deficit less with gastric feeding

14. Acosta-Escribano 2010	Not reported	weight-based	Yes

15. Davies 2012	EN for a median of 8 (interquartile range 5 to 14) days.	Schofield	Yes

Nine studies reported the amount of nutrients administered to patients (calories ± protein). Of these studies, one reported small intestinal feeding reduced the amount of nutrients administered [18], four reported that it increased nutrient delivery [19,20,30,34] and four reported that energy and protein delivered was unaffected by route of feeding [32,33,37] (Table 2).

**Figure 4:**
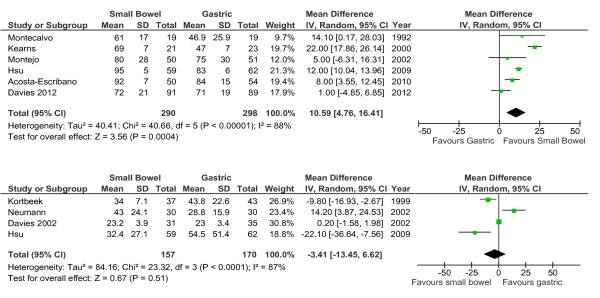
**Small intestinal feeding and nutritional outcomes**. (**A**) Six studies reported nutrient intake, and (**B**) four reported the time taken to reach goal feeding.

Data from six studies that reported nutritional intake as mean ± SD could be aggregated. In these studies there was slight variation in the description of nutrient intake: it was specified as percentage of daily caloric intake, percentage of estimated energy requirements received, and 'mean efficacious volume of diet'. When these data were grouped, small bowel feeding compared to gastric feeding was associated with a significantly greater percentage of nutritional intake (WMD 11% of intake/amount prescribed [5,16]; *P *= 0.0004, I^2 ^= 88%; Figure 4A). Data from studies that reported the time to reach nutritional goal rate were aggregated (*n *= 4), and there was no effect detected (WMD -3.4 hours (-13.5 to 6.6); *P *= 0.51; I^2 ^= 87%; Figure 4B).

## Discussion

We conducted a systematic review and meta-analysis of all RCTs of gastric vs. small bowel feeding in the ICU setting, including the recently published ENTERIC study [21]. The main observations are that delivery of nutrients directly into the small intestine may be associated with a reduction in the incidence of ICU-acquired pneumonia when compared to intragastric delivery, but despite this, days of ventilation, ICU and hospital length of stay and mortality appear unaffected. In addition, while there were relatively few, and substantial heterogeneity between, studies that evaluated nutritional efficiency, there was a signal that feeding into the small intestine increased nutrient intake.

It is possible that our meta-analysis over-estimated any reduction in pneumonia caused by small intestinal feeding. In several studies the diagnosis of pneumonia was made while investigators were aware of treatment allocation. Moreover, nearly all studies were conducted at a single ICU and the number of subjects was relatively small. Biases, including publication and selection biases, are well known to occur in studies involving fewer subjects, thereby affecting point estimates calculated in meta-analyses [38]. To limit bias we also analysed studies that used quantitative microbiological assessment. When this was performed, the interpretation remained similar supporting the original observation. Nevertheless, a reduction in pneumonia was not apparent in the largest cohort studied (ENTERIC). Given that discrepancies between meta-analyses and the 'truth' occur frequently [39,40], circumspect interpretation of these aggregate data related to the incidence of pneumonia is recommended.

While we report a reduction in ICU-acquired pneumonia, the number of days of ventilation, length of ICU and hospital stay, as well as mortality, were unaffected by the route of feeding. While the lack of effect on the latter outcomes may reflect an inadequate sample size or, as described, that our meta-analysis overestimated the effect of route of feeding on the risk of pneumonia, there are plausible mechanisms that may explain these seemingly discrepant findings. In several of the studies artificial nutrition was administered for only a short period and so-called 'early-onset' ventilator-associated pneumonia is often caused by susceptible organisms and responds rapidly to antibiotic therapy [41]. Accordingly, attributed outcomes, such as length of ventilation and mortality, may actually be unaffected by 'early-onset' hospital-acquired pneumonia. Indeed, other factors, such as depth of sedation [42], may be greater determinants of length of ventilation and ICU stay than development of pneumonia.

There was a signal for increased nutritional intake when using small intestinal feeding tubes. There was, however, substantial statistical heterogeneity indicating that this observation should be interpreted with caution. The heterogeneity may reflect that placement of small intestinal tube can be technically difficult, requiring expertise and sophisticated methodologies [25]. Somewhat surprisingly, time-to-placement and placement success was not consistently reported. We suggest that the improvement in nutritional intake will only be generalisable to institutions that have the capacity to rapidly insert feeding tubes into the small intestine.

There were also inconsistencies between studies as to reporting concurrent gastrokinetic drug administration. This is likely to be important when trying to interpret the nutritional data, as gastrokinetic drugs can have potent effects on gastric emptying, but the response varies markedly between drug classes and regimens [4,43,44]. It should, therefore, be emphasised that data relating to nutritional intake do not extend to a comparison between small intestinal feeding and intragastric feeding with concurrent gastrokinetic drug administration.

While nutrient intake may have been 'improved', as discussed, mortality was unaffected. However, the optimal amount of calories and protein that should be administered to the critically ill is uncertain. Moreover, the benefits of nutrient administration may vary according to a number of factors specific to the individual patients. Those likely to benefit more from artificial nutrition compared to other ICU patients are those with a body mass index at either extreme [6], increased NUTRIC score [45], and anticipated prolonged length of stay in the ICU [10]. It should be emphasised that in some studies the period of nutrition required was relatively brief [18]. Hence, the power to detect any benefit from improved nutritional efficiency is markedly diminished [45]. It should also be noted that administration of more nutrient might have effects that are important to patients, but are not measured using data such as length of stay and mortality. Rice and colleagues reported that patients who received fewer calories were less likely to return to independent living on discharge [46]. Unfortunately, only one study reported longer-term function (neurological outcomes in this case) [24] and none measured muscle strength after ICU discharge. These functional outcomes may be very important to patients, and future studies of nutritional interventions would benefit from measuring such outcomes.

A further consideration is that the delivery of nutrients into the small intestine does not guarantee absorption, and it is absorption, rather than delivery of nutrients, which will improve patient outcomes, as undigested nutrients entering into the large intestine will lead to gas formation and abdominal distension, as well as diarrhoea [47]. Whether nutrients are administered proximal or distal to the pylorus does not, however, appear to affect absorption [48].

Although our search strategy was relatively comprehensive, and our methodology robust, there are several limitations to our findings. Statistical heterogeneity was modest and clinical heterogeneity was substantial. Some studies attempted to identify a cohort that was likely to have delayed gastric emptying [21], whereas others evaluated all patients requiring enteral nutrition at the beginning of their ICU stay [18]. There are no studies that include only patients with persistent feed-intolerance and/or those at the greatest risk of ICU-acquired pneumonia. This is important because while delayed gastric emptying occurs frequently in the critically ill [4,5], the prevalence is probably no greater than 30% of all ICU patients that receive enteral nutrition. We suggest that patients with relatively 'normal' gastric emptying and oesophago-gastric motility are unlikely to benefit from small intestinal feeding. For this reason, we recommend against extrapolating this systematic review to patients with documented enteral feed-intolerance (as a marker of delayed gastric emptying) [49], particularly when feed-intolerance occurs during gastrokinetic drug use, and/or those at the greatest risk of ICU-acquired pneumonia - as these groups of patients may well benefit from small intestinal feeding.

Future studies should therefore target patients who develop feed-intolerance while receiving gastrokinetic drugs. In particular, targeting patients who are most likely to benefit from augmented nutritional delivery and who will require nutritional support for a substantial period of time will be a priority. In addition, these studies would also benefit from using a technique that affords rapidly and repeatedly successful placement of small intestinal feeding tubes as well as measuring longer-term functional outcomes.

## Conclusions

Small bowel feeding may be associated with a reduction in ICU-acquired pneumonia and increases in nutrient delivery, but days of ventilation, ICU and hospital stay, and mortality were unaffected. Until further data are available, decisions as to whether to preferentially feed patients into the small intestine will need to be at an institutional level, incorporating the feasibility, safety and delays in obtaining access, while identifying patients most likely to benefit from this route of feeding.

## Key messages

• In the critically ill, small intestinal feeding when compared to intragastric may reduce the incidence of ICU-acquired pneumonia.

• In the critically ill, small intestinal feeding when compared to intragastric may increase nutritional intake.

• The route of enteral nutrient administration (intragastric or small intestinal) does not appear to be a major determinant of mortality or length of stay in unselected critically ill patients.

## Abbreviations

CI: Confidence interval; ICU: Intensive care Unit; PRISMA: Preferred Reporting Items for Systematic Reviews and Meta-Analyses; RCT: Randomised control trial; RR: Relative risk; SD: Standard deviation; WMD: Weighted mean difference.

## Competing interests

The authors declare that they have no competing interests.

## Authors' contributions

AD contributed to acquisition, analysis and interpretation of data, and was responsible for drafting, editing and submission of the manuscript. RD contributed to study conception and design. She was responsible for acquisition, analysis and interpretation of data and contributed to revision of the manuscript for important intellectual content. ADay contributed to study conception and design, as well as acquisition, analysis and interpretation of data. ER contributed to study conception and design, as well as acquisition, analysis and interpretation of data. ADavies contributed to study conception and design, acquisition, analysis and interpretation of data, and revision of the manuscript for important intellectual content. DH was responsible for conception and design, acquisition, and revising the manuscript for important intellectual content. All authors have read and approved the final manuscript.
