# Comparisons between intragastric and small intestinal delivery of enteral nutrition in the critically ill: a systematic review and meta-analysis

**DOI:** 10.1186/cc13961

**Published:** 2014-07-01

**Authors:** Adam M Deane, Rupinder Dhaliwal, Andrew G Day, Emma J Ridley, Andrew R Davies, Daren K Heyland

**Affiliations:** 1 Intensive Care Unit, Level 4, Emergency Services Building, Royal Adelaide Hospital, North Terrace, Adelaide 5000, South Australia, Australia; 2 Discipline of Acute Care Medicine, University of Adelaide, Level 5, Eleanor Harrald Building, Frome Road, Adelaide 5000, South Australia, Australia; 3 Clinical Evaluation Research Unit, Kingston General Hospital, Kingston K7L 2 V7, Ontario, Canada; 4 Department of Epidemiology and Preventive Medicine, Monash University, The Alfred Centre, Level 6, 99 Commercial Road, Melbourne 3004, Victoria, Australia

## 

After publication of this article [1], the authors noticed that their names were listed incorrectly as M Deane Adam, Dhaliwal Rupinder, G Day Andrew, J Ridley Emma, R Davies Andrew and K Heyland Daren. The author names should be written as Adam M Deane, Rupinder Dhaliwal, Andrew G Day, Emma J Ridley, Andrew R Davies and Daren K Heyland.
